# Ryanodine receptor 2 promotes colorectal cancer metastasis by the ROS/*BACH1* axis

**DOI:** 10.1002/1878-0261.13350

**Published:** 2022-12-21

**Authors:** Tianwei Chen, Xilin Zhang, Xufen Ding, Jing Feng, Xueli Zhang, Dong Xie, Xiang Wang

**Affiliations:** ^1^ Key Laboratory of Integrated Oncology and Intelligent Medicine of Zhejiang Province, Department of Hepatobiliary and Pancreatic Surgery Affiliated Hangzhou First People's Hospital, Zhejiang University School of Medicine Hangzhou China; ^2^ Department of Central Laboratory First Affiliated Hospital of Huzhou University China; ^3^ CAS Key Laboratory of Nutrition, Metabolism and Food Safety, Shanghai Institute of Nutrition and Health, Shanghai Institutes for Biological Sciences Chinese Academy of Sciences Shanghai China; ^4^ Department of General Surgery Fengxian Hospital Affiliated to Southern Medical University Shanghai China

**Keywords:** colorectal cancer, metastasis, ROS, *RyR2*

## Abstract

There is no targeted therapy for *KRAS* proto‐oncogene, GTPase (*KRAS*)‐mutant metastatic colorectal cancer (mCRC) because the underlying mechanism remains obscure. Based on bioinformatic analysis, this study aims to elucidate a potential gene target for which an approved drug is available, and to reveal the function as well as the underlying mechanism of the candidate gene. Here, we identified that ryanodine receptor 2 (*RyR2*) expression was upregulated in *KRAS*‐mutant mCRC, and that this promoted cancer cell metastasis. S107, an approved drug to inhibit calcium release from *RyR2* in the clinic, inhibited cancer cell metastasis both *in vitro* and *in vivo*. High expression of *RyR2* predicts poor survival in our patient cohort. CRC patients with serosa invasion and vascular tumor thrombus are characterized by high *RyR2* expression. Analysis of expression profiles upon *RyR2* knockdown and inhibition, revealed a set of metastasis‐related molecules, and identified BTB domain and CNC homolog 1 (*BACH1*) as the main transcription factor regulated by *RyR2*. *RyR2* regulates cellular reactive oxygen species (ROS) levels, which activates nuclear factor erythroid 2‐related factor 2 (*Nrf2*; also known as *NFE2L2*) and *HMOX1* expression, and thus *BACH1* accumulation. Collectively, this study provides evidence that the *RyR2*/ROS/*BACH1* axis may be a potential intervention target for CRC metastasis.

## Graphical abstract

Through bioinformatics analysis combined with molecular strategies, *RyR2* was identified as a regulator of colorectal cancer metastasis. *RyR2* expression raised intracellular Ca^2+^ concentration and promoted cellular ROS levels, which in turn activated Nrf2 activity followed by *BACH1* accumulation. The *BACH1* accumulation led to the start‐up of the metastasis progression of cancer cells.

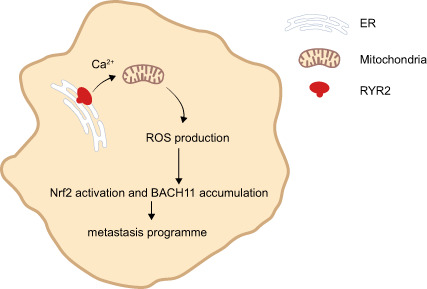

**Abbreviations**
[Ca^2+^]_i_
: intracellular calcium concentration
ARE: antioxidant response elements
*BACH1*
: BTB and CNC homology 1
CPVT: catecholaminergic polymorphic ventricular tachycardia
CRC: colorectal cancer
DAB: 3,3′‐diaminobenzidine
DCFH: 2,7‐dichlorodihydrofluorescein diacetate
DEG: deregulated genes
ECM: extracellular matrix
EGFR: EGF receptor
ER: endoplasmic reticulum
FOLFIRI: folinic acid, fluorouracil and irinotecan
FOLFOX: folinic acid, fluorouracil and oxaliplatin
HE stain: hematoxylin and eosin stain
HMOX1: heme oxygenase 1
HR: hazard ratio
IF: immunofluorescence
IHC, immunohistochemistry
mitoSOX: mitochondrial superoxide
Nrf2: nuclear factor erythroid 2‐related factor 2
PLC: phospholipase C
ROS: reactive oxygen species
*RyR2*
: ryanodine receptor 2
SOCE: store operated calcium entry
TBS: Tris‐buffered saline
TCGA: The Cancer Genome Atlas
TF: transcription factor
TMA: tissue microarray

## 1 Introduction

Colorectal cancer (CRC) represents one of the most common digestive tract malignancies worldwide [1]. Half the diagnosed cases will develop into metastatic colorectal cancer (mCRC), which is the final step of cancer development and the main cause of CRC‐related death [2]. Given the pervasive activation of the EGF receptor (EGFR) signaling pathway in CRC and mCRC, the EGFR antibodies cetuximab and panitumumab are approved to treat mCRC together with the traditional chemotherapy including folinic acid, fluorouracil and irinotecan (FOLFOX) and folinic acid, fluorouracil and oxaliplatin (FOLFIRI) [3]. However, mCRC patients with *KRAS* activation mutation (generally at codons 12 and 13) are excluded from this treatment modality, resulting in a poor prognosis [4]. An appealing discovery of AMG510 as a specific inhibitor of *KRAS*G12C in 2019 is encouraging, although this type of mutation represents only 3% of CRC patients [5]. Thus proper intervention and treatment are still both urgently needed for this subset of mCRC patients. However, this is hindered by a lack of knowledge of the mechanisms underlying metastasis formation of *KRAS* mutant patients.

Calcium regulates diverse biological processes including cancer metastasis [5]. An early study showed that *STIM*/*ORAI*, which mediates extracellular calcium fluxing into endoplasmic reticulum (ER) to refill the intracellular calcium reservoir, is critical for breast cancer metastasis [6]. The next decade witnessed numerous studies demonstrating the crucial role of calcium in cancer progression, which involves a great number of molecules, including calcium channels, transporters as well as calcium binding proteins, and diverse pathways regulated by intracellular calcium levels [7]. However, the cellular outputs triggered by calcium changes are complex due to both the pattern of calcium stimulation and the cellular calcium toolbox. To elicit a calcium‐related signal, extracellular stimuli converge on calcium channels residing in ER [8]. ER‐resident calcium channels consist of two families, the inositol triphosphate receptors 1–3 (*IP3R1‐3*) and the ryanodine receptors 1–3 (*RYR1‐3*) [9]. *IP3R* bind intracellular inositol 1,4,5 triphosphate (IP3), the end product of phospholipase C (PLC), and release calcium from ER to cytosol [10]. *RyR* show a tendency for expression in the brain, heart and muscle, where they sense membrane potential changes [11]. As such, *IP3R* are considered functional in non‐excitable cells, whereas *RyR* are mainly involved in excitable cells, including nerve and muscle cells.

In this study, we found that *RyR2* was upregulated in *KRAS* mutant mCRC patients, and *RyR2* high expression conferred poor survival. *RyR2* gene silencing by knocking down or pharmacological inhibition by small molecule S107 decreased cancer cell metastasis both *in vitro* and *in vivo*. A mechanism study uncovered genes downstream of *RyR2*, and enriched *BACH1* as the main transcription factor (TF) affected by *RyR2* inhibition. Further research revealed that *RyR2* regulated *BACH1* levels via modulating cellular reactive oxygen species (ROS) level. Collectively, the results of this study provide the therapeutic possibility that *RyR2* targeting by S107 may be repurposed to intervene with metastasis of CRC patients.

## 2 Materials and methods

### 2.1 Cell culture

HEK293T and SW480, HCT116, HT29 and CT26 were purchased from Cell Bank of Type Culture Collection of Chinese Academy of Sciences, Shanghai Institute of Cell Biology, Chinese Academy of Sciences. All cells were grown in DMEM (Gibco, Carlsbad, CA, USA) supplemented with 10% FBS (Anlite, Shanghai, China) and 1% penicillin/streptomycin (Sangon Biotech, Shanghai, China), except for CT26 cells, which was cultured with RPMI‐1640 (Gibco). Cell lines were incubated at 37 °C in a humidified 5% CO_2_ atmosphere.

### 2.2 CRC sample and tissue microarray

A total of 195 patients diagnosed pathologically with colorectal cancer were enrolled in this study. Samples were collected at The First People's Hospital of Huzhou from 2018 to 2019 in compliance with the protocol for tissue collection approved by the Ethics Committee of First Affiliated Hospital, Huzhou University (approval number: 2020KYLL002). All methodologies conformed to the standards set by the Declaration of Helsinki. Informed consent was signed by all patients and all experiments were approved by the Ethical Committee. Detailed pathological examination parameters were recorded and available for analysis. Tissue microarray (TMA) was constructed from CRC samples embedded in paraffin, and stained with anti‐*RYR2* (diluted 1 : 100), followed by scanning, photographing and scoring using the Vectra2 system (PerkinElmer, Waltham, MA, USA).

### 2.3 RNA isolation and real time PCR

RNA extraction and cDNA preparation have been described earlier [12]. Briefly, total RNA was isolated using TRIzol reagent. Total RNA 2 μg was transcribed to cDNA with a reverse transcription kit (Promega, Madison, WI, USA). Real‐time PCR was performed using SYBR premix Taq (Yeasen Biotech, Shanghai, China) in an Mx3000P Real‐Time detection system (Stratagene, La Jolla, CA, USA). Primers are listed in the Supplemental Table S2.

### 2.4 Immunohistochemistry and immunofluorescence

The procedures of these two assays have been described earlier [12]. Anti‐*RYR2* (Proteintech) was diluted 1 : 100 to stain CRC TMA slides. Anti‐phospho‐*CREB*(Ser133) (Affinity, Changzhou, China) was diluted 1 : 100 in an immunofluorescence (IF) assay. Briefly, for immunohistochemistry (IHC), sections of clinical specimens were deparaffinized with xylene and rehydrated with ethanol, followed by staining with anti‐*RYR2* antibody and horseradish peroxidase (HRP)‐linked anti‐rabbit IgG, and further developed with 3,3′‐diaminobenzidine (DAB). TMA sections were further photographed and analyzed by Vecture 2 (PerkinElmer). The same algorithm was used to score every core. For IF, cells on slides were fixed in 4% formaldehyde, followed by staining with indicated primary antibodies and fluorescent secondary antibody (Alexa Fluor 488‐conjugated donkey anti‐rabbit IgG and Alexa Fluor 555‐conjugated goat anti‐rabbit IgG, 1 : 1000) and were photographed with a confocal microscope (Zeiss, Cambridge, UK; LSM 880NLO FILM).

### 2.5 Plasmids and stable cell lines

The pLKO.1 was used to produce shRNA lentivirus and pHAGE‐fEF1a‐IRES‐ZsGreen was used to produce lentivirus carrying *TIAM2*. For stable cell line production, CRC cell lines were transfected with lentivirus for 48 h along with polybrene (1 μg·mL^−1^), followed by GFP sorting (pHAGE‐fEF1a‐IRES‐ZsGreen vector) or puromycin treatment (pLKO.1 vector; 3 days and longer).

### 2.6 Western blot and immunoprecipitation

Western blot analysis was performed as described previously [12]. 3xFlag‐*BACH1* immunoprecipitation was performed according to the anti‐Flag M2 manual. Briefly, 2 days post transfection, HEK293T cells were washed twice in ice‐cold PBS, and lysed in lysis buffer (50 mm Tris–HCl, pH = 7.4 with 150 mm NaCl, 1 mm EDTA and 1% Triton X‐100) with protease inhibitors for 10 min on ice, after centrifugation at 20 000 *g* for 15 min at 4 °C. Supernatants were incubated with anti‐Flag M2 beads on a rotator overnight in cold room. After incubation, the beads were pelleted and washed five times in TBS (50 mm Tris–HCl, 150 mm NaCl, pH = 7.4), then elution with 3xFlag peptides for 1 h. The eluate was resolved by SDS/PAGE western blot.

### 2.7 *In vitro* migration assay

The migration assay was performed as described previously [12]. *In vitro* migration assay was conducted in 24‐well inserts (Corning Inc., Corning, NY, USA). Briefly, when cells reached a confluence of 70–90%, they were trypsinized, and 1 × 10^6^ cells were seeded on the top and complete medium was added to the bottom. After incubation for 48 h, non‐invasive cells were removed from the upper end of the insert with a cotton swab. The bottom cells (invasive cells) were fixed with 4% paraformaldehyde for 20 min, stained with a 0.1% crystal violet solution for 30 min, and photographed using a microscope. The number of cells was counted, and data were presented as the means of three randomly selected fields.

### 2.8 RNA‐seq analysis of transcriptome

A total of 3 × 10^6^ SW480 cells were seeded into 10‐cm dishes at two replicates; for the S107 treatment group, the final S107 concentration was 10 μm. Two days later, cells were washed with ice‐cold PBS three times, followed by dissociation in 1 mL TRIzol reagent. Samples were further subjected to RNA extraction, library construction and high‐throughput sequencing (Illumina NovaSeq; Illumina Inc., San Diego, CA, USA). Raw data were quality controlled and mapped to the genome by HISAT2, followed by transformation to FPKM. DESeq was used to calculate deregulated genes (DEG) with¦log_2_FoldChange¦ > 1 and *P* < 0.05. To obtain DEG between *RYR2* ^low^ and *RYR2* ^high^ in the Cancer Genome Atlas (TCGA) data, we first scored the mRNA expression data of TCGA‐COAD and TCGA‐READ using the r package estimate to acquire immune and stroma score for every patient. We chose the relative pure tumor tissues by selecting total score < −500 and immune score < −100. Respectively, 22 and 12 patients were grouped into *RYR2* ^low^ and *RYR2* ^high^. DEG were obtained using the r package limma. All heatmap pictures were drawn by the r package pheatmap. TF prediction was performed using the DAVID website.

### 2.9 Dual luciferase reporter assay

Reporter sequences for *BACH1*, *CHX10*, *FREAC7*, *HFH3* and *SOX9* were synthesized and inserted into backbone plasmid; sequences for each TF can be found in the Supplemental Table S2. To perform dual luciferase reporter assay, 0.1 μg reporter plasmid and 0.02 Renilla luciferase vector were transformed into cells using lipo3000 reagent for three replicates. Two days after transfection, the measurements were complied using the instruction manual.

### 2.10 Animal studies

BALB/c mice (Shanghai SLAC Laboratory Animal Co. Ltd., Shanghai, China) were housed according to a 12‐h light (7 a.m.)/dark (7 p.m.) cycle at 25 °C, with *ad libitum* access to water and rodent standard chow diet and maintenance under pathogen‐free conditions. All animal studies were approved by and performed in accordance with Institutional Animal Care and Use Committee of Shanghai Institutes for Nutritional Sciences, Chinese Academy of Sciences (SIBS‐2018‐XD‐3). Metastatic murine colorectal cancer cell line CT26 was used in this study. For the weekly luciferase reporter assay, mice were injected intraperitoneally with d‐luciferin. They were anesthetized by isoflurane and photographed in the IVIS imaging system (Xenogen, Alameda, CA, USA).

For lung metastasis, 4 × 10^5^cells were injected into the tail vein of each nude mouse. Two weeks post injection, all mice were sacrificed and lungs were subjected to fixation in formalin followed by embedding in paraffin and then H&E staining.

For splenic injection, 5 × 10^5^cells were injected into mouse spleen. S107 was used 2 days before the surgery and was injected intraperitoneally every other day at a concentration of 30 mg·kg^−1^. Ten days post injection, the mice were sacrificed and their livers excised.

### 2.11 Heme detection

Cell‐free heme was determined by the colorimetric method using a commercially available kit (MAK316‐1KT; Sigma‐Aldrich, St. Louis, MO, USA). Briefly, cells in a 10‐cm dish with a confluence of about 80% were washed with ice‐cold PBS, followed by collection of cells with a scrapper. Cells were pelleted and resuspended in lysis buffer (PBS with protease inhibitor), followed by cellular disruption by sonication. The remaining steps followed those in the manufacturer's manual.

### 2.12 Statistical analysis

All data are presented as the mean ± standard error of the mean. Student's *t*‐test was used for the comparison of measurable variants of two groups. All experiments were performed with at least three biological duplicates (*n* = 3) for each group. Survival curves were calculated using the Kaplan–Meier method, and differences were assessed by a log‐rank test. The criterion for significance was *P* < 0.05 for all comparisons.

## 3 Results

### 3.1 *RyR2* upregulation is related to colorectal cancer metastasis behavior

**Fig. 1:**
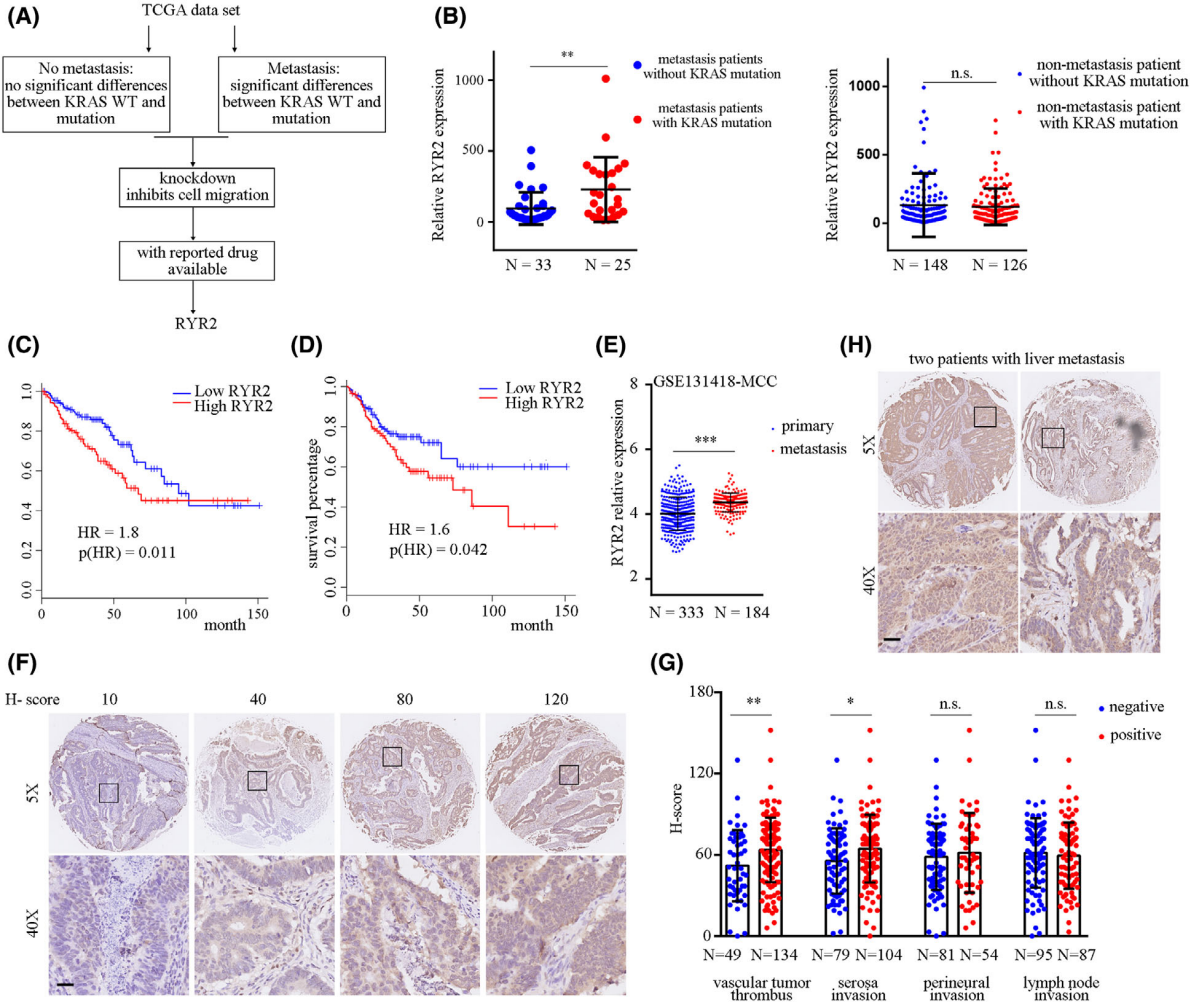
*RYR2* expression was associated with CRC metastasis behavior. (A) Schematic workflow showing strategy to identify target gene. (B) Dot plots demonstrating mRNA expression of *RYR2* in different groups of patients. **P < 0.01; n.s., not significant. (C,D) Line plots showing result of Kaplan–Meier analysis of survival percentage in different patient groups. HR, hazard ratio. (E) Dot plot demonstrating mRNA expression of *RYR2* in different tumor tissues. ***P < 0.001. (F) Images exhibiting signal intensities and their corresponding IHC scores. A total of 195 images were scored. Scale bar: 50 μm. (G) Dot plot showing *H*‐score in different groups. *P < 0.05; **P < 0.01; n.s., not significant. (H) Images showing *RYR2* staining intensities of two CRC patients with liver metastases. Scale bar: 50 μm. Data are presented as the mean ± standard error of the mean. Student's *t*‐test was used for the comparison of measurable variants of two groups.

To identify differentially expressed genes in *KRAS* mutant metastatic CRC patients, we analyzed TCGA CRC data using the following strategy. Patients were first divided into two groups according to their metastasis status. For each group, patients were further separated by *KRAS* status. Candidate genes should be upregulated in *KRAS* mutant mCRC patients compared with *KRAS* WT mCRC patients, but showed no significant changes in the non‐metastasis group. These criteria yielded 13 genes. The candidate genes were further screened by shRNA in the SW480 cell line which showed *KRAS* G12V mutation, followed by examination of migration ability *in vitro*. We then examined whether there was a targeted drug available for the candidate genes which led to *RyR2* as the final target (Fig. 1A). *RyR2* showed upregulation in *KRAS* mutant mCRC patients compared with *KRAS* WT patients, but its expression was not changed in non‐metastasis patients (Fig. 1B). To gain insights into the clinical significance of *RyR2* upregulation, we analyzed TCGA CRC data and demonstrated that high expression of *RyR2* in CRC patients resulted in a shorter survival time (hazard ratio [HR] = 1.8, *P* = 0.011; Fig. 1C) as well as shorter disease‐free survival (HR = 1.6, *P* = 0.042; Fig. 1D). Consistently, in another cohort comprising 575 patients, we also observed a shorter overall survival time associated with high *RYR2* expression (Fig. S1A). Kamal and colleagues reported the largest transcriptome data of mCRC and primary CRC tissues [12]. By analyzing this dataset, we found that *RyR2* expression was significantly higher in mCRC tissues than in primary CRC tissues (Fig. 1E).

To further characterize the correlation between *RyR2* and CRC metastasis behavior, we stained a TMA consisting of 195 primary CRC tissues. Scoring for each tissue core was accomplished by Vectra 2 and was further manually curated (Fig. 1F). Statistical analysis of IHC score with clinicopathologic parameters showed that *RyR2* expression was not correlated with tumor size (*P* = 0.114), P53 status (*P* = 0.279) or Ki67 status (*P* = 0.731), indicating that *RyR2* expression did not affect tumor growth (Table S1). Four metastasis‐associated parameters were included in our cohort: serosa invasion, vascular tumor thrombus, perineural invasion and lymph node invasion. Perineural invasion and lymph node invasion were not correlated with *RyR2* expression. Importantly, *RyR2* expression was significantly upregulated in CRC patients with tumor serosa invasion (*P* = 0.0049) and intratumoral vascular tumor thrombus (*P* = 0.0155) (Fig. 1G). Furthermore, in the two patients enrolled in our cohort who manifested liver metastases, high *RyR2* expression could be observed in the primary tissues from both patients (Fig. 1H).

Taken together, high *RyR2* expression in primary CRC tissue was correlated with serosa invasion and vascular tumor thrombus formation, and predicted a poor prognosis. The mCRC tissues showed elevated *RyR2* expression compared with the primary CRC tissues. For mCRC patients, *RyR2* was overexpressed in *KRAS* mutant subtype compared with *KRAS* WT patients.

### 3.2 *RyR2* knockdown or inhibition decreased CRC cell metastasis

**Fig. 2:**
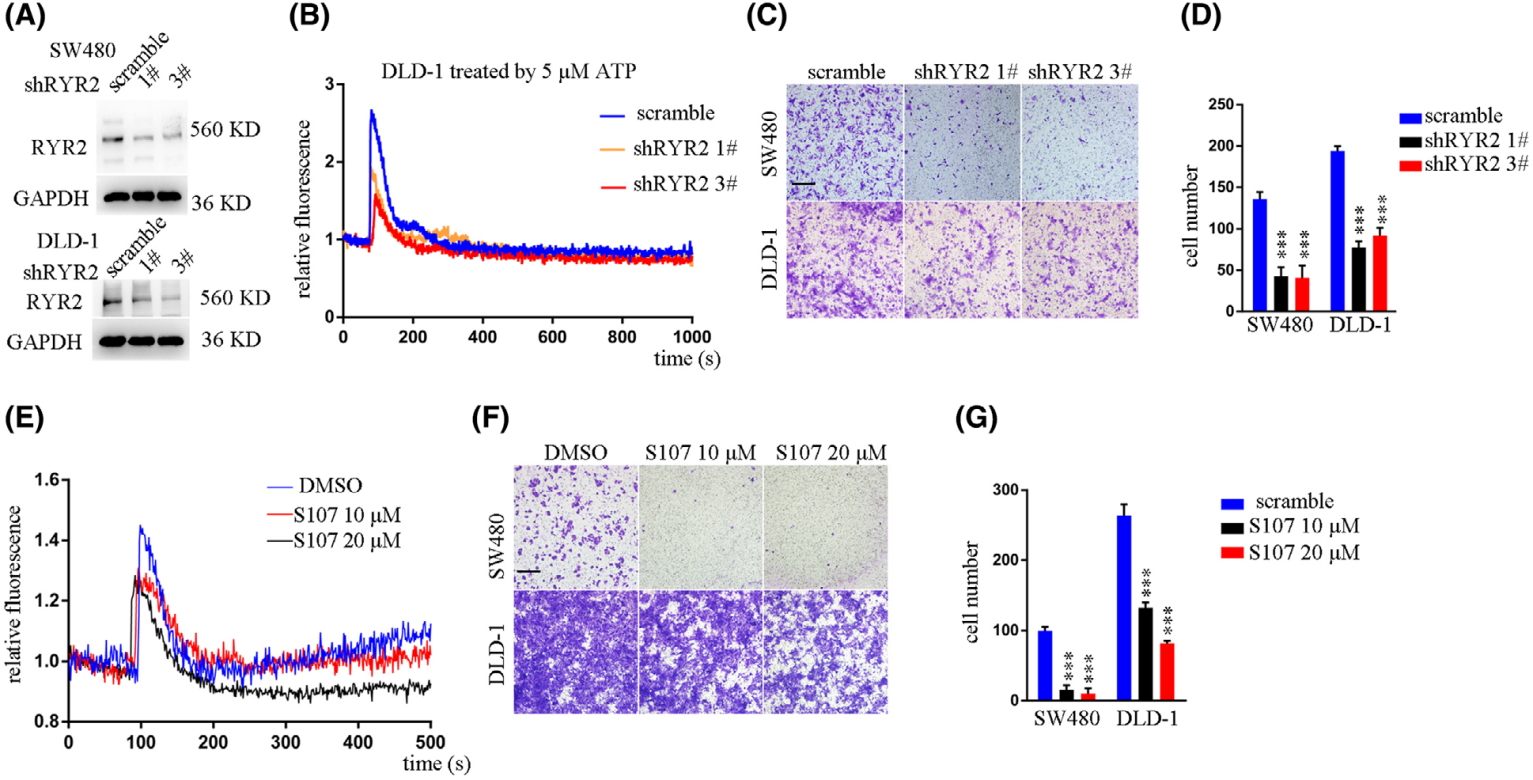
*RYR2* KD or inhibition decreased CRC migration *in vitro*. (A) Western blot results showing *RYR2* KD efficacy. (B) Line plot exhibiting fluorescence changes (Fluo 4 signal) in indicated cells of different groups. (C) Representative images showing results of Transwell assay. Scale bar: 200 μm. (D) Bar plot showing statistical results of Transwell assay. ***P < 0.001. (E) Line plot exhibiting fluorescence changes (Fluo 4 signal) in indicated cells of different groups. (F) Representative images showing result of Transwell assay. Scale bar: 200 μm. (G) Bar plot showing statistical results of Transwell assay. ***P < 0.001. SW480 and DLD‐1, two human colorectal cancer cell lines. ATP, adenosine triphosphate. S107, a chemical inhibitor of RyR2 calcium channel. Data are presented as the mean ± standard error of the mean. Student's *t*‐test was used for the comparison of measurable variants of two groups. All experiments were performed with at least three biological duplicates (*n* = 3) for each group, in triplicate.

To demonstrate the cellular function of *RyR2*, we first knocked down *RyR2* expression using two independent shRNA sequences in *KRAS* mutant CRC cell line DLD‐1 (*KRAS*G13V), SW480 (*KRAS*G12V) and HCT116 (*KRAS*G13D) (Fig. 2A; Fig. S1B). *RyR2* is a critical molecule in the calcium‐induced calcium release (CICR) process, and ATP has been shown to increase cellular calcium. We tested whether ATP‐induced intracellular calcium increase was inhibited by *RyR2* knockdown (KD). ATP 5 μm substantially increased intracellular free calcium [Ca^2+^]_i_ in DLD‐1 control cells, while this [Ca^2+^]_i_ increase was hindered after *RyR2* KD, although *RyR2* KD did not affect the responsive time point and maintenance time window (Fig. 2B). Transwell assay demonstrated significant inhibition of cellular migration ability after *RyR2* KD (Fig. 2C,D). While we found that *RyR2* KD still inhibited cellular migration in a *KRAS* wildtype cell line RKO, the inhibitory extent was limited to 10%, compared with more 50% in SW480 and DLD‐1 (Fig. S1C).

S107 is a small molecule that enhances calstabin2 binding to *RyR2* (R2474S) and is orally available to prevent cardiac arrhythmias and raise the seizure threshold [13]. We found that S107 treatment in SW480 cells dampened [Ca^2+^]_i_ enhancement triggered by 5 μm ATP (Fig. 2E). Consistently, S107 treatment inhibited SW480 and DLD‐1 cell migration (Fig. 2F,G). Further examination of cellular growth revealed that *RyR2* KD or its inhibition by S107 did not affect CRC cell growth (Fig. S1D–G).

**Fig. 3:**
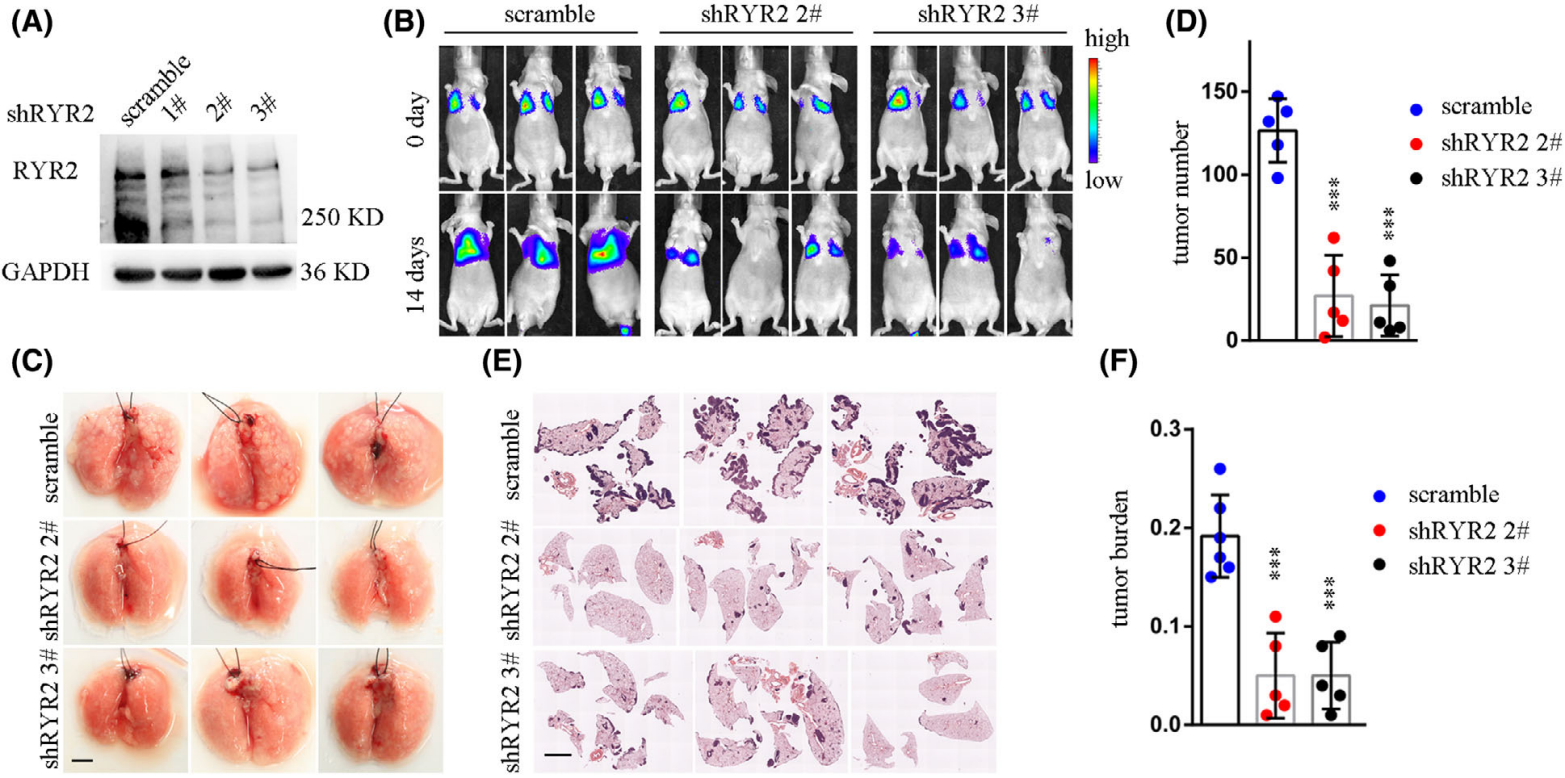
*RYR2* KD inhibited CRC cell metastasis *in vivo*. (A) Western blot result showing efficacy of *RYR2* KD in CT26‐luci cells. (B) Representative bioluminescent images demonstrating signal intensities of different groups. (C) Bright‐field pictures exhibiting lungs of mouse with metastasis model. Scale bar: 5 mm. (D) Scatter plot with bar showing tumor foci number in different groups. ***P < 0.001. (E) H&E staining of lungs. Scale bar: 4 mm. (F) Scatter plot with bar showing tumor burden in different groups. ***P < 0.001. Data are presented as the mean ± standard error of the mean. Student's *t*‐test was used for the comparison of measurable variants of two groups. All experiments were performed with at least three biological duplicates (*n* = 3) for each group, in triplicate.

We next asked whether *RyR2* KD or inhibition could decrease CRC cell metastasis *in vivo*. To this end, we used the highly metastatic CRC cell line CT26, which carried *KRAS*G12D mutation. We first knocked down the expression of *RyR2* in CT26‐luciferase cells (Fig. 3A). Delivery of cancer cells into mice vasculature via tail vein injection developed a strong signal in lungs at 14 days post injection. However, *RyR2* KD resulted in a significant decrease in signal magnitude (Fig. 3B). Consistently, whereas hundreds of metastatic foci were observed on the surfaces of lungs from control mice, lungs from the *RyR2* KD groups presented with much fewer metastatic foci (Fig. 3C); statistical analysis demonstrated an average of 126 ± 8 metastatic foci in the control group, and 27 ± 10 and 21 ± 8 metastatic foci for the two sh*RyR2* groups, respectively (Fig. 3D). Whole‐mount HE staining of lungs revealed a higher tumor burden in the control group compared with the sh*RyR2* groups (Fig. 3E,F).

**Fig. 4:**
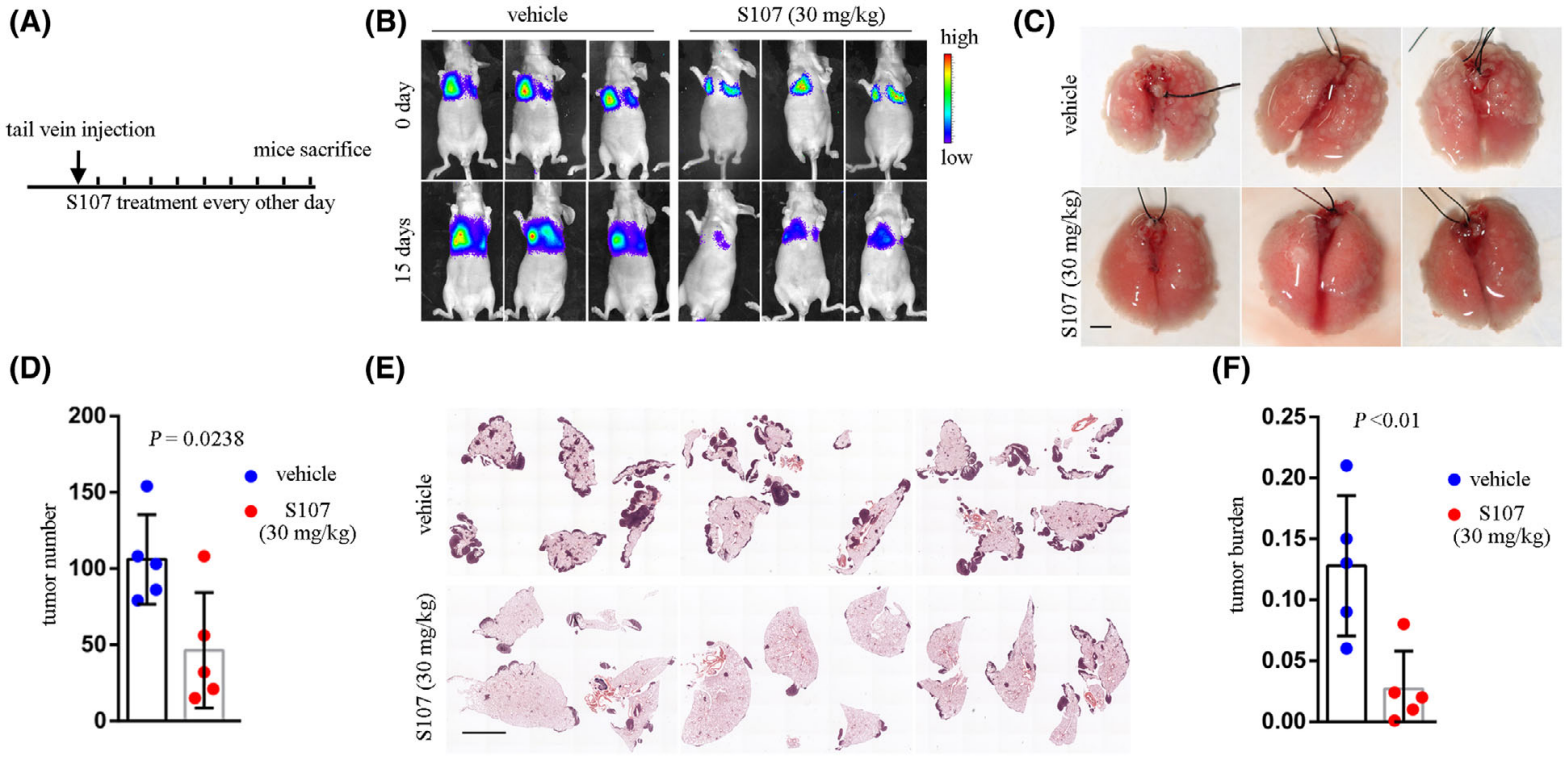
S107 inhibited CRC cell metastasis *in vivo*. (A) Schematic diagram showing experimental procedure. (B) Representative bioluminescent images demonstrating signal intensities of different groups. (C) Bright‐field pictures exhibiting lungs of mouse with metastasis model. Scale bar: 5 mm. (D) Scatter plot with bar showing tumor foci number in different groups. Statistical *P*‐value shown. (E) H&E staining of lungs. Scale bar, 5 mm. (F) Scatter plot with bar showing tumor burden in different groups. Statistical *P* value shown. Data are presented as the mean ± standard error of the mean. Student's *t*‐test was used for the comparison of measurable variants of two groups. All experiments were performed with at least three biological duplicates (*n* = 3) for each group, in triplicate.

We next sought to examine the therapeutic effect of S107 in impeding CRC metastasis. CT26‐luci cells were pretreated with S107 for 2 days before being injected into mice tail veins, followed by S107 treatment at 30 mg·kg^−1^ via intraperitoneal injection every other day (Fig. 4A). Bioluminescent images showed significantly decreased signals in the S107 treatment group compared with the control group (Fig. 4B). Furthermore, S107 treatment reduced the number of metastasis foci when compared with the control group (Fig. 4C,D), and also attenuated tumor burden (Fig. 4E,F). Moreover, using splenic injection of CT26 cells to model CRC liver metastasis, we observed decreased metastasis formation following S107 treatment (Fig. S2). Taken together, *RyR2* inhibition decreased CRC cell metastasis both *in vitro* and *in vivo*.

### 3.3 *RyR2* regulates a set of metastasis‐associated genes

**Fig. 5:**
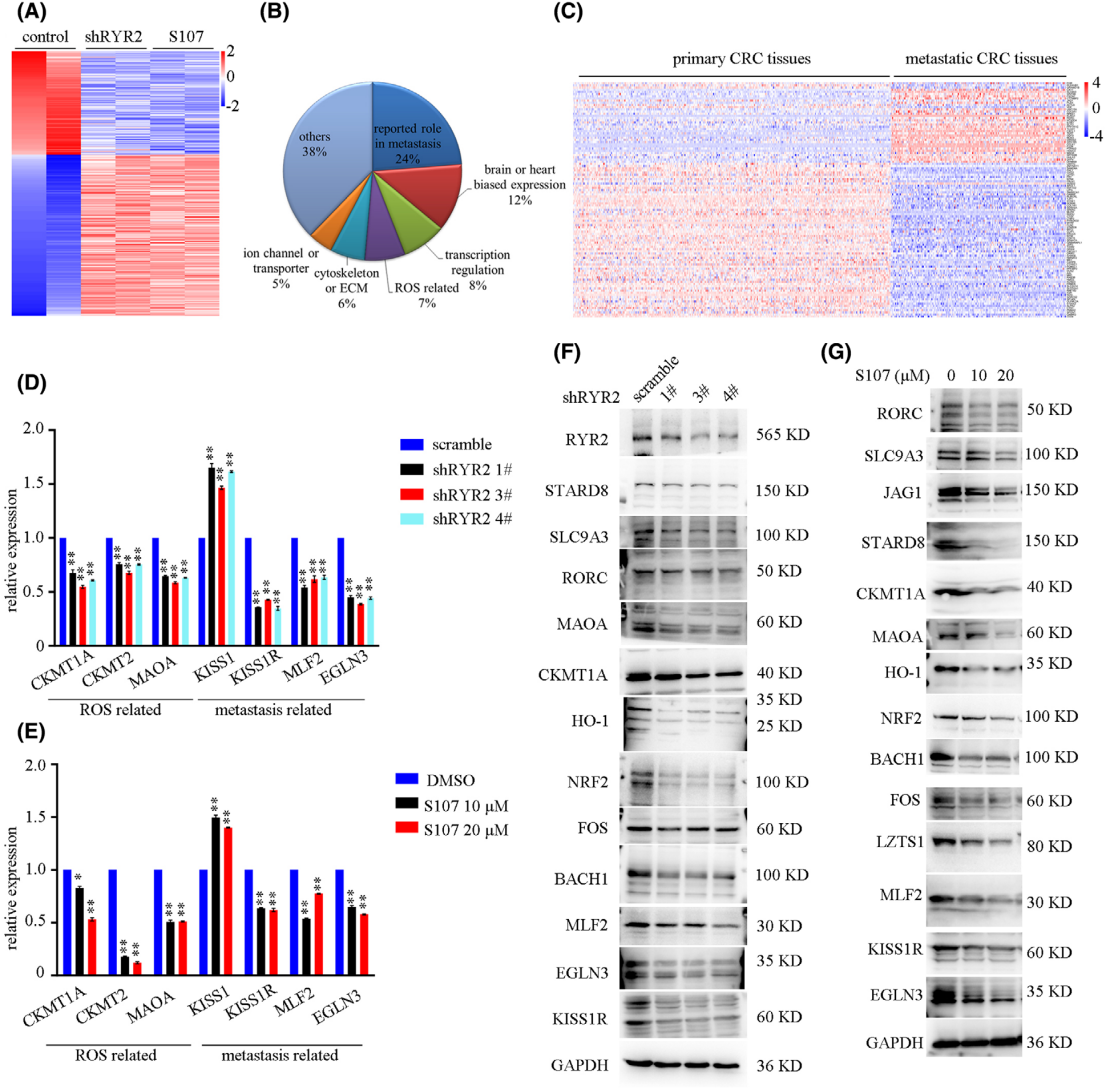
*RYR2*‐regulated genes. (A) Heatmap showing differential expressed genes up *RYR2* KD or inhibition. (B) Sector graph showing enrichment of DEG. DEG, deregulated genes. (C) Heatmap demonstrating differential expression of *RYR2* regulated genes in primary and metastasis CRC tissues. CRC, colorectal cancer. (D,E) Bar plot demonstrating mRNA expression in different groups. *P < 0.05; **P < 0.01. (F, G) Western blot results showing protein expression in different groups. Data are presented as the mean ± standard error of the mean. Student's *t*‐test was used for the comparison of measurable variants of two groups. All experiments were performed with at least three biological duplicates (*n* = 3) for each group, in triplicate.

To gain insights into the molecular changes after *RyR2* KD or S107 treatment, we performed RNA‐seq analysis. The result demonstrated 112 co‐downregulated genes and 174 co‐upregulated genes following *RyR2* KD or S107 treatment (Fig. 5A). A manual check of these 286 deregulated genes (*RyR2* associated DEG) revealed enrichment in cancer metastasis (68 genes), brain‐ or heart‐biased expression (35 genes), transcription regulation (23 genes), ROS related function (21 genes), cytoskeleton or extracellular matrix (ECM) (18 genes), ion channel or transporters (13 genes) (Fig. 5B). To further identify functional genes during colorectal cancer metastasis, we analyzed gene expression of these 286 DEG in primary and metastatic CRC tissues by *in silico* analysis of previously reported data (GSE131418). To this end, we identified 37 genes downregulated by *RyR2* KD and S107 treatment that showed a higher expression in metastatic CRC tissues, and 71 genes upregulated by *RyR2* KD or S107 treatment that showed a lower expression in metastatic CRC tissues (Fig. 5C).

We then validated the RNA‐seq results by RT‐PCR; the results showed that expressions of the representative molecules such as ROS‐related *CKMT1A*, *CKMT2* and *MAOA* as well as metastasis‐associated *KISS1*, *KISS1R*, *MLF2* and *EGLN3* were consistent with our RNA‐seq results (Fig. 5D,E). Moreover, Western blot (WB) result also confirmed the RNA‐seq results (Fig. 5F,G). These data revealed a set of metastasis‐associated genes downstream of *RyR2*.

### 3.4 Transcription factor *BACH1* was regulated by *RyR2*

**Fig. 6:**
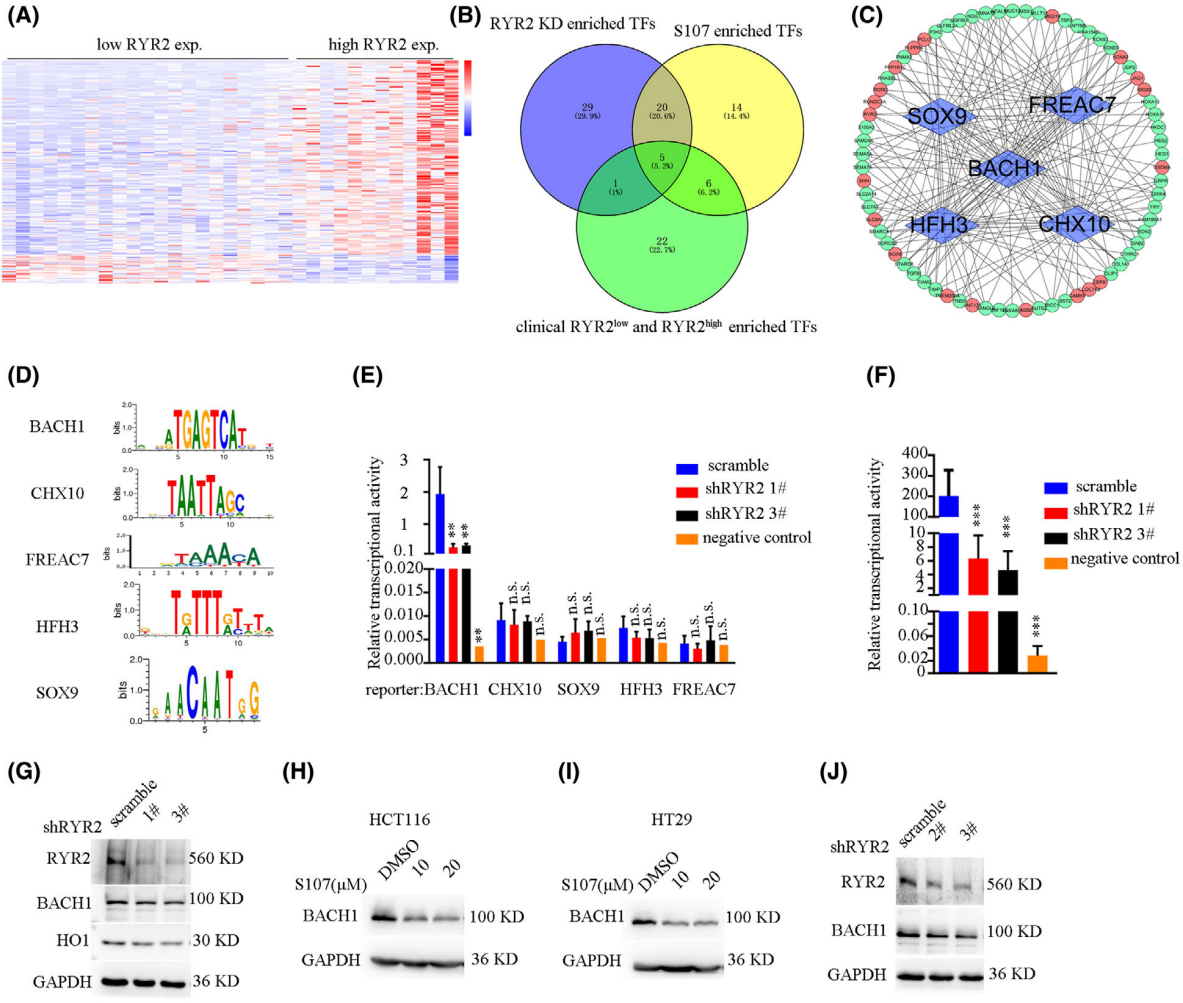
Transcription factor was downstream of *RYR2*. (A) Heatmap showing differentially expressed genes in *RYR* ^*low*^ and *RYR* ^*high*^ groups. (B) Venn diagram exhibiting transcription factors enriched by differentially expressed genes. TF, transcription factors. (C) Circle map showing transcription factors and their corresponding target genes. (D) Images showing binding sites of different transcription factors. (E) Bar plot showing relative luciferase activity of different transcription factors. **P < 0.01. n.s., not significant. (F) Bar plot showing relative luciferase activity of HCT116 cells. ***P < 0.001. (G–J) WB result showing that *RYR2* or S107 treatment reduced *BACH1* levels in different CRC cell lines. Data are presented as the mean ± standard error of the mean. Student's *t*‐test was used for the comparison of measurable variants of two groups. All experiments were performed with at least three biological duplicates (*n* = 3) for each group, in triplicate.

As *RyR2* regulated a large number of metastasis‐related genes, we proposed that *RyR2* might reshape cell fate. TF controlled cell fate by regulating the expression of multiple genes. We thus asked whether there existed any TF whose activities were modulated by *RyR2*. To this end, we took advantage of three lines of evidence: (i) TF enriched by DEG following *RyR2* KD; (ii) TF enriched by DEG following S107 treatment; (iii) TF enriched by DEG derived from TCGA data, by comparing low and high *RyR2* expression samples (Fig. 6A). We thus enriched 55, 45 and 34 TF for these three groups, respectively. Five TF emerged as shared by all three groups (Fig. 6B,C). To further verify this result, we first constructed luciferase reporter plasmids for the five TF according to their binding sites (Fig. 6D). Dual luciferase reporter assay showed high basal level transcriptional activity of *BACH1* and low activities of the other four TF. Moreover, *RyR2* KD significantly decreased *BACH1* reporter activity (Fig. 6E). *RyR2* KD induced a decrease in *BACH1* reporter activity that could also be observed in HCT116 cells (Fig. 6F). In an attempt to explore the underlying mechanism, we found that *RyR2* KD or inhibition by S107 downregulated the protein level of *BACH1* (Fig. 6G) in SW480 cells. The results could also be reproduced in HCT116 cells (Fig. 6H) as well as in HT29 cells and CT26 cells (Fig. 6I,J). Thus, *BACH1* was regulated by *RyR2*.

### 3.5 *RyR2*‐induced ROS production and cellular heme level

**Fig. 7:**
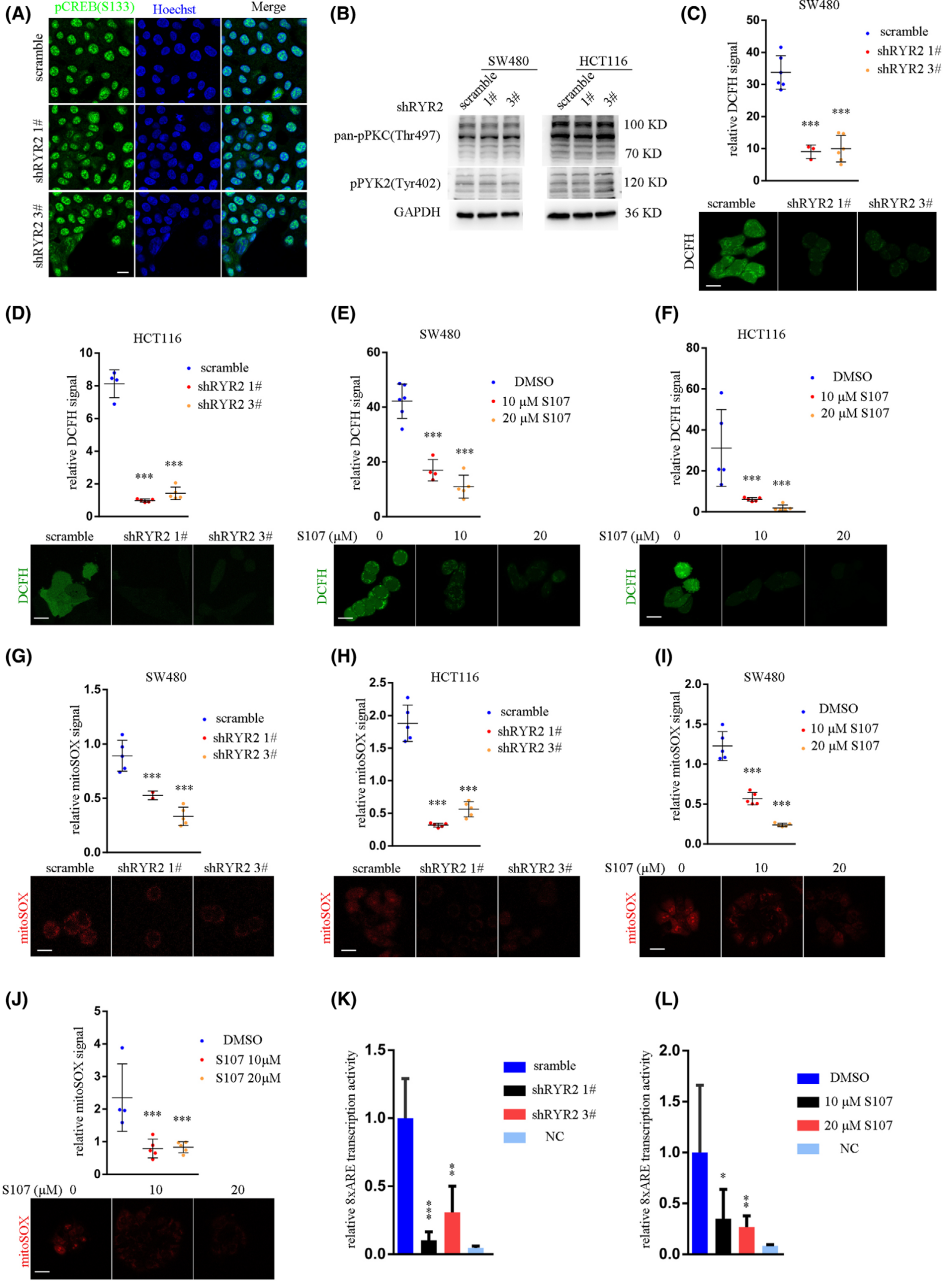
*RYR2*‐regulated cellular ROS production. (A) Representative images showing localization of pCREB(S133). Scale bar: 50 μm. (B) WB result showing pPKC(Thr497) and pPYK2(Tyr402) after *RYR2* KD. (C–F) Dot plot and images showing intensity of cellular DCFH signal in SW480 cells (C,E) and HCT116 cells (D,F). ***P < 0.001. DCFH, 2,7‐dichlorodihydrofluorescein diacetate. Scale bar: 25 μm. (G–J) Dot plot and images showing intensity of cellular mitoSOX signal in SW480 cells (G,I) and HCT116 cells (H,J). Scale bar: 25 μm. (K,L) Bar plot demonstrating 8xARE reporter activity of *RYR2* KD (K) and S107 treatment (L). mitoSOX, mitochondrial superoxide; ARE, antioxidant response elements. **P < 0.01. Data are presented as the mean ± standard error of the mean. Student's *t*‐test was used for the comparison of measurable variants of two groups. All experiments were performed with at least three biological duplicates (*n* = 3) for each group, in triplicate.

We next asked how *RyR2* KD decreased *BACH1* expression. As *RyR2* was an ER‐resident calcium channel, we first checked whether classical pathways regulated by calcium were changed by *RyR2* KD. Excitation‐transcription coupling described transcriptional activity regulated by calcium‐p*CREB* axis in cardiac myocytes. However, p*CREB*(Ser133) was not changed following *RyR2* KD in SW480 cells (Fig. 7A). Another important cellular aspect regulated by calcium was *PKC* signaling pathway. In SW480 cells, *RyR2* KD did not affect either p*PKC*(Thr497) or p*PYK2*(Tyr402) (Fig. 7B). In addition, *BACH1* did not interact with known molecules downstream of calcium (calmodulin, *CaMKIV*, *CaMKII*) (Fig. S3A). We identified an array of proteins interacting with *BACH1* using proximity proteomics, and selected some proteins whose functions were closely related to calcium; however, none of these proteins interacted with *BACH1* (Fig. S3B). Thus, we inferred that *RyR2* influenced *BACH1* expression in a more indirect manner.

Previous studies on *BACH1* revealed its regulation by antioxidants and *Nrf2* [14, 15] which were related to cellular ROS. This and our RNA‐seq results led us to determine cellular ROS changes following *RyR2* KD or inhibition. *RyR2* KD did indeed significantly decrease cellular ROS level in both SW480 and HCT116 cells (Fig. 7C,D). Consistently, S107 treatment also reduced ROS levels in both cell lines (Fig. 7E,F). Cellular ROS was mainly derived from superoxide species, which were inevitably produced by oxidative phosphorylation in mitochondria. *RyR2* KD led to a significant decrease in superoxide species in SW480 and HCT116 cells, as measured by mitoSOX signal (Fig. 7G,H). S107 treatment also inhibited superoxide species production in SW480 and HCT116 cells (Fig. 7I,J). As a result, *RyR2* KD or inhibition by S107 downregulated the *Nrf2* level (Fig. 5F,G). Consequently, antioxidant response element (ARE) reporter activity was significantly dampened by *RyR2* KD or inhibition (Fig. 7K,L). As previously reported, transcriptional upregulation of heme oxygenase 1 (*HMOX1*) by *Nrf2*‐mediated *BACH1* degradation. We also found downregulation of *HMOX1* expression after *RyR2* KD or inhibition (Fig. 5F,G). Since *BACH1* directly regulated the cellular heme level, we measured free heme levels in SW480 and DLD‐1 cells with *RyR2* KD. The results showed that *RyR2* KD significantly enhanced cellular heme levels (Fig. S4). Taken together, *RyR2* KD or inhibition decreased cellular ROS levels, which in turn inactivated *Nrf2* activity, and thus *HMOX1* downregulation and heme accumulation followed by *BACH1* degradation.

### 3.6 Genes downstream of *RyR2*/*BACH1* affected cellular motility

**Fig. 8:**
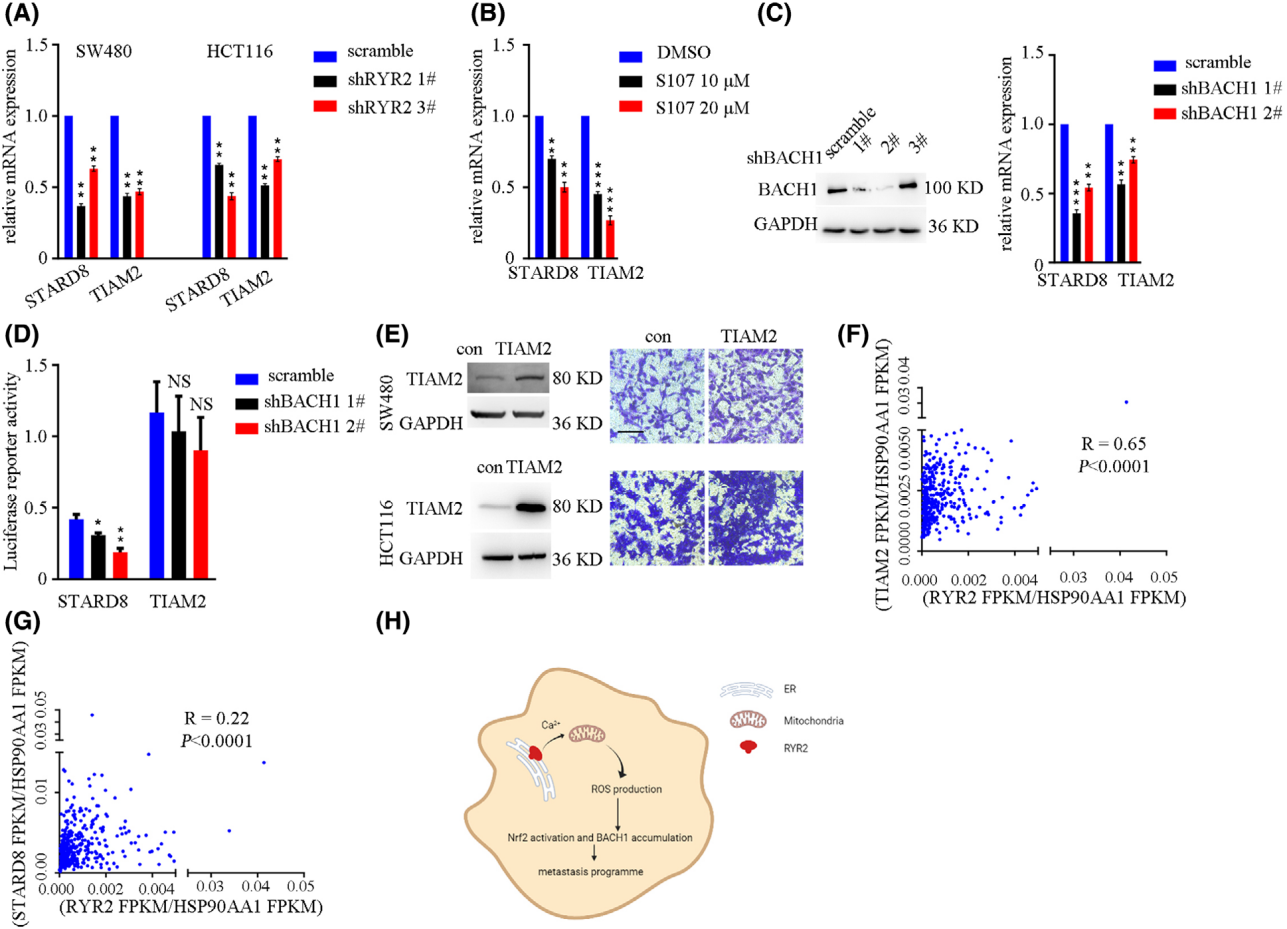
*STARD8* and *TIAM2* were downstream target genes of *RyR2*. (A) Bar plot showing mRNA expression of target genes in cells. **P < 0.01. (B) Bar plot showing mRNA expression of target genes in cells. **P < 0.01. (C) Western blot result showing *BACH1* KD efficacy (left). Bar plot showing mRNA expression of target genes in cells. **P < 0.01. (D) Bar plot showing relative luciferase reporter activity in cells. *P < 0.05; **P < 0.01. NS, not significant. (E) Western blot showing *TIAM2* overexpression efficacy (left). Representative images showing Transwell result. Scale bar: 50 μm. (F) Dot plot showing the correlation of *RYR2* expression and *TIAM2* in TCGA dataset. (G) Dot plot showing the correlation of *RYR2* expression and *STARD8* in TCGA dataset. (H) Diagram showing the hypothesis of this work. Data are presented as the mean ± standard error of the mean. Student's *t*‐test was used for the comparison of measurable variants of two groups. All experiments were performed with at least three biological duplicates (*n* = 3) for each group, in triplicate.

*BACH1* is well established as a pro‐metastasis TF. However, different *BACH1* downstream targets have been reported in certain kinds of cancer types. Combining *RyR2* DEG and putative targets predicted by JASPAR, we identified a gene list of *BACH1* downstream genes, which were further exemplified by its putative targets *STARD8* and *TIAM2*. Both *RyR2* KD and inhibition by S107 downregulated mRNA levels of *STARD8* and *TIAM2* (Fig. 8A,B). *BACH1* KD in HCT116 cells also hindered mRNA expression of *STARD8* and *TIAM2* (Fig. 8C). Furthermore, *BACH1* expression in 293T cells enhanced promoter activity of *STARD8*. However, this regulation was not observed for the promoter of *TIAM2* (Fig. 8D). Instead, the *BACH1* binding site was detected at the enhancer region of *TIAM2*, as indicated in GeneCards by Hi‐C sequencing data. We next overexpressed *TIAM2* in SW480 and HCT116 cells (Fig. 8E). The results demonstrated that overexpression of *TIAM2* enhanced cellular motility (Fig. 8E). Moreover, mRNA expression of *STARD8* and *TIAM2* was highly correlated with that of *RyR2* in TCGA‐COAD dataset (Fig. 8F,G). Thus, the *RyR2*/*BACH1* axis regulated *STARD8* and *TIAM2* in order to regulate CRC cell motility.

## 4 Discussion

There is still an urgent need for appropriate therapeutic modality for mCRC patients with *KRAS* mutation. To identify druggable genes that can mediate metastasis of this group of patients, we combined bioinformatic analysis, molecular and cell biology and found that *RyR2* and its inhibitor S107 possessed the potential to be repurposed to intervene with *KRAS* mutant CRC metastasis. *RyR2* was upregulated in mCRC patients with *KRAS* mutation and its expression was associated with poor prognosis of CRC patients. Moreover, its high expression was associated with serosa invasion and tumor vascular thrombus. This is evidence that *RyR2* expression in tumor cells was closely related to metastasis behavior of CRC. Previous studies on *RyR2* were mainly focused on the physiological and pathological roles in heart and brain, where *RyR2* was highly expressed; few studies have reported its role in cancer metastasis [16, 17].

Multiple congenital *RyR2* mutations cause calcium leakage from ER and lead to catecholaminergic polymorphic ventricular tachycardia (CPVT) in humans [18]. S107 was first reported to inhibit calcium leakage from mutant *RyR2* through enhancement of the binding affinity between *RyR2* and calstabin2 [13]. We found that S107 actually inhibited calcium leakage in SW480 and DLD‐1 cells with WT *RyR2*. The ability of S107 to inhibit cancer cell metastasis and ROS production was consistent with the results of *RyR2* KD. Furthermore, mice treated with S107 at 30 mg·kg^−1^ via intraperitoneal injection for 2 weeks presented with a healthy appearance. As S107 was commercially available, we wondered whether this drug could be repurposed to intervene with CRC metastasis.

Calcium has long been linked to cancer metastasis [19]. One of the early studies shows that *Orai1* and *STIM1*, which mediate extracellular calcium influx into ER via store‐operated calcium entry (SOCE), were critical for breast cancer cell metastasis [6]. That study highlighted the essential role of ER‐resident calcium in cancer metastasis. Calcium release from ER to cytosol was mainly governed by six members (*RyR1‐3* and *IP3R1–3*) [9]. Although these six channels share a similar function, it is intriguing to find that only high *RyR2* expression predicts a poor prognosis in CRC (Fig. S5). High *RyR2* expression in cardiomyocytes leads to spontaneous Ca^2+^ leakage from ER [20], and subsequent diminished systolic Ca^2+^ transients which are not necessary for cancer cells. Effectors downstream of *RyR2* include *NF‐κB*/*cyclin D1* in pulmonary arterial smooth muscle cells [21], glucose oxidative phosphorylation [22] and ROS production by calcium entry into mitochondria matrix in cardiomyocytes [23], Interferon β (IFNβ) signaling in 293T and monocytes among others [24]. The discovery here supports the model that *RyR2* overexpression increases superoxide species production in mitochondria and subsequent ROS levels in cytosol, followed by *KEAP1* inactivation, and *Nrf2* nuclear translocation and *BACH1* accumulation (Fig. 8H).

*BACH1* is well established as a promoter of cancer metastasis, especially in non‐small cell lung cancers with *NFE2L2* mutation [14, 15]. Evidence also exists showing that *BACH1* promotes CRC cell metastasis [25, 26, 27]. Generally, *BACH1* is a member of the Cap ‘n’ Collar and basic region leucine zipper family of TF and it acts as a transcription repressor of target genes [28]. We found that *RyR2* KD decreases the cellular *BACH1* level, and thus dysregulation of *BACH1*‐regulated metabolism and metastasis genes. *BACH1* does not interact with canonical calcium‐related molecules, including calmodulin, *CaMKII* and *CaMKIV*, indicating that *BACH1* is not a first‐line effector of cellular calcium change. The crosstalk between calcium and *BACH1* sheds further light on the regulation of cancer metastasis by calcium.

Intimate relationships between cancer metastasis and mitochondrial activity, ROS and *Nrf2* are frequently reported [29, 30, 31, 32, 33]. Targeting calcium is also recognized as a potential option in cancer therapy [19]. However, considering the huge amounts of molecules involved in these pathways, it is not easy to identify a specific druggable gene in a specific cancer type.

## 5 Conclusions

Through bioinformatics analysis and experimental assays, our results provide evidence that *RyR2* is a potential therapeutic target for intervention of CRC metastasis.

## Conflict of interest

The authors declare no conflict of interest.

## Author contributions

TC, XW conceived the project. TC, XiZ performed most of the experiment. XD conducted some molecular experiments. JF analyzed some data. XuZ, DX, XW supervised this study. TC wrote the report.

### Peer review

The peer review history for this article is available at https://publons.com/publon/10.1002/1878‐0261.13350.

## Supporting information


**Fig. S1.** RyR2 inhibition did not affect cellular growth.

**Fig. S2.** RyR2 inhibition decreased CRC liver metastasis *in vivo*.

**Fig. S3.** RyR2 did not interact with calcium‐related molecules.

**Fig. S4.** RyR2 inhibition increased cellular heme level.

**Fig. S5.** Overall survival and disease‐free survival analysis of ITPR and RYR.

**Table S1.** Relationship between expression of RYR2 in CRC and clinicopathologic features.

**Table S2.** Materials and primers used in this study.

## Data Availability

The data that support the findings of this study are available from the corresponding author at wangxiang2021@zju.edu.cn upon reasonable request.
