# Supplementary figures and images for: Ryanodine receptor 2 promotes colorectal cancer metastasis by the ROS/BACH1 axis
Source: Mol Oncol. 2022 Dec 21;17(4):695–709. doi: 10.1002/1878-0261.13350 (PMC10061290; doi:10.1002/1878-0261.13350)

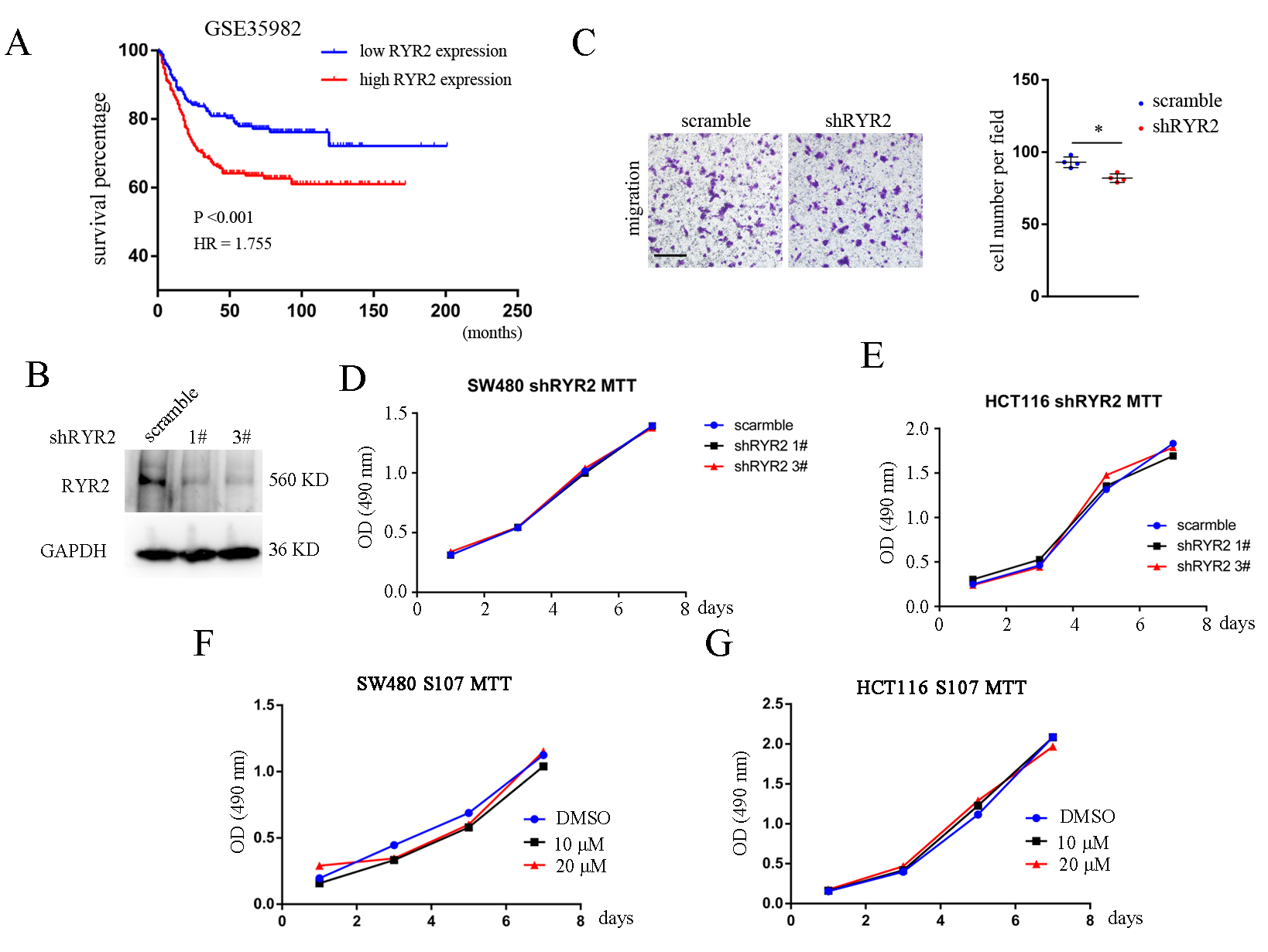

Supplement: Supplementary file 1 — Fig. S1. RyR2 inhibition did not affect cellular growth. [file MOL2-17-695-s006.jpg]

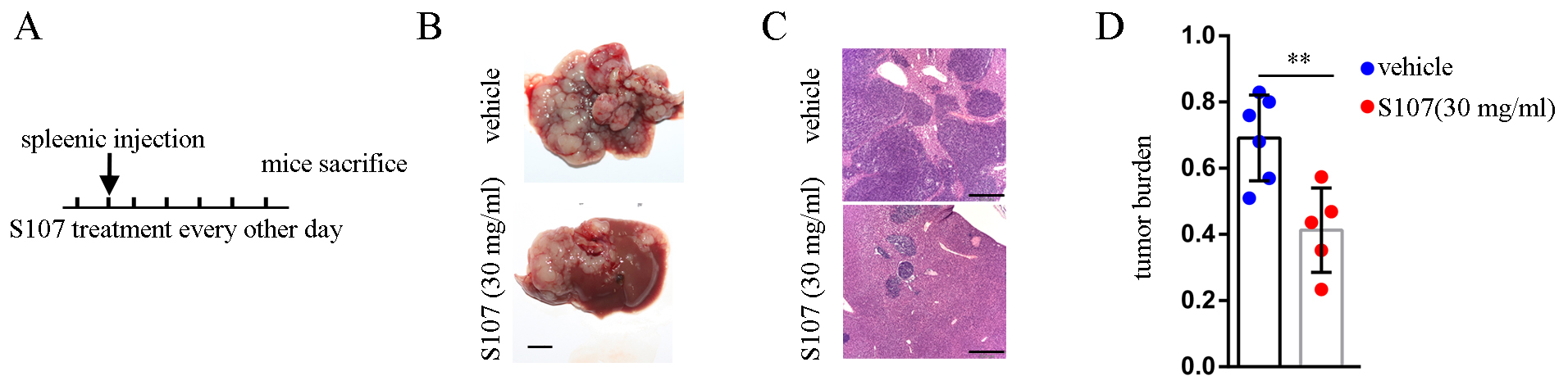

Supplement: Supplementary file 2 — Fig. S2. RyR2 inhibition decreased CRC liver metastasis in vivo. [file MOL2-17-695-s002.jpg]

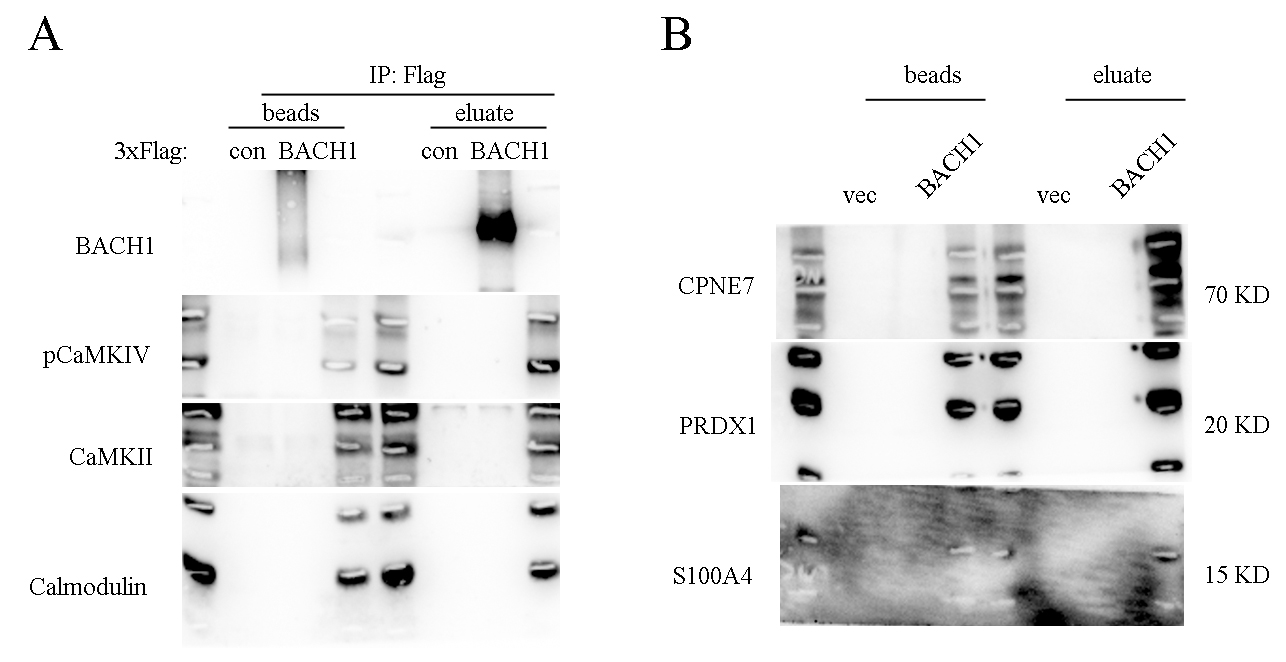

Supplement: Supplementary file 3 — Fig. S3. RyR2 did not interact with calcium‐related molecules. [file MOL2-17-695-s003.jpg]

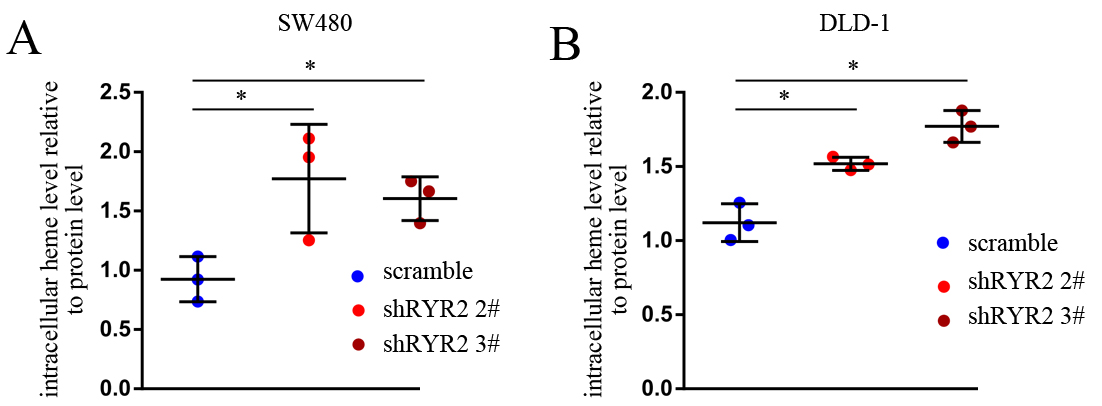

Supplement: Supplementary file 4 — Fig. S4. RyR2 inhibition increased cellular heme level. [file MOL2-17-695-s001.jpg]

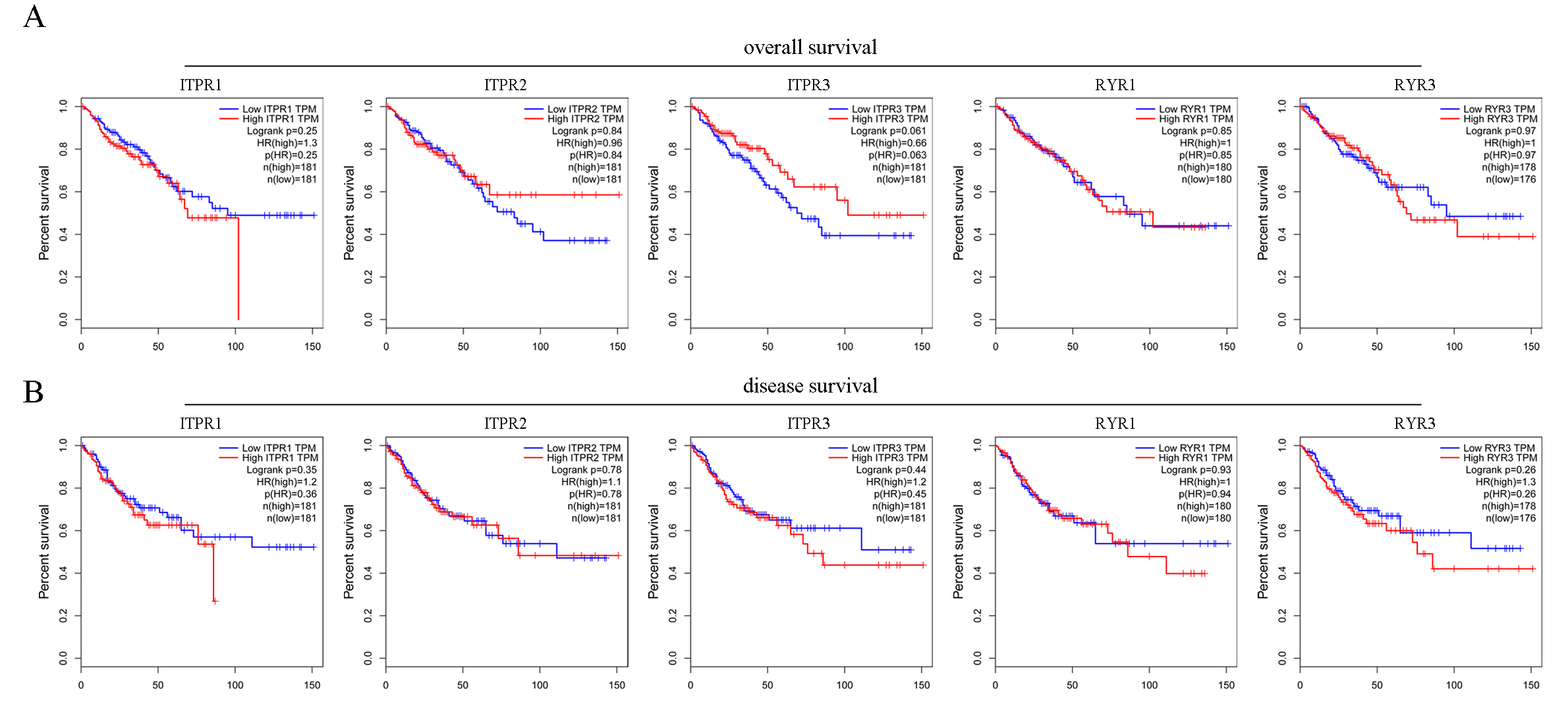

Supplement: Supplementary file 5 — Fig. S5. Overall survival and disease‐free survival analysis of ITPR and RYR. [file MOL2-17-695-s004.jpg]
