# Supplementary material for: Ryanodine receptor 2 promotes colorectal cancer metastasis by the ROS/BACH1 axis
Source: Mol Oncol. 2022 Dec 21;17(4):695–709. doi: 10.1002/1878-0261.13350 (PMC10061290; doi:10.1002/1878-0261.13350)
Supplement: Supplementary file 6 — Table S1. Relationship between expression of RYR2 in CRC and clinicopathologic features. [file MOL2-17-695-s007.docx]

**RyR2 promotes colorectal cancer metastasis by the ROS/*BACH1* axis**

Chen *et al.*

Supplemental materials: 1 table, 5 figures..

**Supplemental Table 1** Relationship between expression of RYR2 in CRC and clinicopathologic features

| Characteristic^a^ | Low Expression Group | High Expression Group | χ^2^ | *P* value |
| --- | --- | --- | --- | --- |
| **Tumor size** |  |  |  |  |
| < 5 cm | 54 | 58 | 2.493 | 0.114 |
| ≥ 5 cm | 27 | 47 |  |  |
| **P53 status** |  |  |  |  |
| < 20% | 41 | 43 | 1.170 | 0.279 |
| ≥ 20% | 40 | 58 |  |  |
|  |  |  |  |  |
| **Ki67 status** |  |  |  |  |
| weak | 5 | 6 | 1.290 | 0.731 |
| weak to medium | 6 | 11 |  |  |
| Medium to strong | 24 | 34 |  |  |
| strong | 47 | 51 |  |  |

^a^ some characteristics were not recorded for some patients.

**Supplemental Figure 1 RyR2 inhibition did not affect cellular growth.** (A) Kaplan-Meier survival plot of survival possibility in GSE35982. (B) Western blot result showing RyR2 KD efficacy. (C) Representative image showing the result of transwell. *, *P*<0.05. Scale bar, 100 μm. (D-G) Line plots showing MTT results of shRYR2 in SW480 cells (D) and HCT116 cells (E) as well as showing MTT result of S107 treatment of SW480 cell (F) and HCT116 cells (G).

**Supplemental Figure 2 RyR2 inhibition decreased CRC liver metastasis in vivo.** (A) Schematic drawing showing workflow of the experiment. (B) Representative image showing liver from experimental mouse. Scale bar, 1 cm. (C) Representative HE image showing the magnified liver conditions. Scale bar, 500 μm. (D) Dot plot showing tumour burden of each mouse. **, *P*<0.01.

**Supplemental Figure 3 RyR2 did not interact with calcium-related molecules.** (A&B) Western blot showing immunoprecipitation results of Flag-BACH1 in 293T cells.

**Supplemental Figure 4 RyR2 inhibition increased cellular heme level.**  Dot plot showing heme level in different cells in SW480 (A) and DLD-1 (B). *, *P*<0.05.

**Supplemental Figure 5 Overall survival and Disease-free survival analysis of ITPRs and RYRs.** (A) Line plots of Kaplan-Meier overall survival analysis results of indicated genes in TCGA colorectal cancer dataset. (B) Line plots of Kaplan-Meier disease-free survival analysis results of indicated genes in TCGA colorectal cancer dataset.
